# Detection of Macrolide-Resistant *Streptococcus pneumoniae* Genes and Its Clinical Outcomes in a Tertiary Teaching Hospital in Malaysia

**DOI:** 10.21315/mjms2024.31.2.17

**Published:** 2024-04-23

**Authors:** Wan Norliyana Wan Mahmud, Siti Asma’ Hassan, Zaidah Abd Rahman, Wan Nor Amilah Wan Abdul Wahab, Nabilah Ismail

**Affiliations:** 1 Hospital Kemaman, Terengganu, Malaysia; 2 Department of Medical Microbiology and Parasitology, School of Medical Sciences, Universiti Sains Malaysia, Kelantan, Malaysia; 3 School of Health Sciences, Universiti Sains Malaysia, Kelantan, Malaysia

**Keywords:** macrolide, resistant, genes, Streptococcus pneumoniae, susceptibility

## Abstract

**Background:**

*Streptococcus pneumoniae* is one of the leading causes of mortality and morbidity worldwide. The dramatic increase in in-vitro resistance of antimicrobial agents, particularly beta-lactams and macrolides, makes pneumococcal infections difficult to treat. The aim of this study was to describe the drug resistance rate, assess the prevalence of macrolide-resistant genes and review the clinical complications of pneumococcal infections among patients presented to Hospital Universiti Sains Malaysia (HUSM), Kelantan, Malaysia.

**Methods:**

This is a descriptive cross-sectional study. All *S. pneumoniae* isolates collected from clinical specimens within a 1-year period were subjected to selected antimicrobial susceptibility testing using E-test strips. Polymerase chain reaction (PCR) analysis was conducted to detect macrolide-resistant determinants. The patient’s clinical data were obtained from clinical notes.

**Results:**

A total of 113 patients with a positive growth of *S. pneumoniae* were included in the study. The most common predisposing factors among them were bronchopulmonary diseases (15.9%). The penicillin-resistant rate was 7.1%, with minimal inhibitory concentration (MIC) ranging between 0.012 μg/mL and >32 μg/mL, and the erythromycin-resistant rate was 26.5%, with a MIC range of 0.03 μg/mL–> 256 μg/mL. Most of the erythromycin-resistant isolates were found to have the *mef*(A) gene (50.4%) and the *erm*(B) gene (20%); 16.7% had a combination of genes *mef*(A) and *erm*(B), and 13.3% had none of the two genes. Community-acquired pneumonia is the predominant type of pneumococcal infection. There was no significant association between the presence of macrolide resistance determinants and mortality (*P* = 0.837) or complications (*P* > 0.999 for empyema and cardiac complication; *P* = 0.135 for subdural abscess).

**Conclusion:**

The majority of erythromycin-resistant isolates were found to have the *mef*(A) gene, followed by the *erm*(B) gene and a combination of genes *mef*(A) and *erm*(B).

## Introduction

*Streptococcus pneumoniae* is one of the leading causes of mortality and morbidity worldwide, posing a major public health problem. It causes more serious infections in infants under 2 years old (1) and adults older than 65 years old (2). *S. pneumoniae* is a Gram-positive bacteria spread by airborne droplets, and it is a primary cause of bacterial pneumonia, acute otitis media, sinusitis, bacteremia and meningitis. The World Health Organization (WHO) estimates that, in the Asia Pacific region, 49 out of 98 cases of pneumonia deaths in children are due to pneumococcal pneumonia. Several studies reported that pneumonia is the most common pneumococcal disease in Malaysia (3, 4). Additionally, about 4% of the 7,000 deaths among children less than 5 years old were thought to be caused by *S. pneumoniae* (5).

The dramatic increase in the prevalence of antibiotic resistance, especially against first-line antibiotics such as penicillin and macrolide, makes pneumococcal infections difficult to treat, especially in children and the elderly (6). In some Asian countries, the reported prevalence of penicillin and macrolide resistance was the highest in the world (7, 8). Previous surveillance studies showed that more than 60% of pneumococcal isolates from Asian countries were resistant to erythromycin and 22.7% were penicillin-resistant (9). In China, erythromycin resistance rates for *S. pneumoniae* increased from 35% to 53% in 1996–1999, to over 75% by 2001, and South Korean resistance rates against erythromycin remained high from 1996 to 2001 (75%–85%). While for Malaysia, 32% were resistant to erythromycin in the year 2012 (9).

Ribosomal methylation encoded by *erm*(B) gene was the most common mechanism of erythromycin resistance in China, Taiwan, Sri Lanka and Korea (7). While in Hong Kong, Singapore, Thailand and Malaysia, an efflux pump encoded by the *mef*(A) gene was more common. *Erm*(B) gene was found in more than 50% of pneumococcal isolates either alone or in combination with *mef*(A) gene in most Asian countries except Hong Kong, Malaysia and Singapore (7). Although antibiotic resistance among *S. pneumoniae* has been increasingly noted worldwide, the clinical significance of in-vitro resistance is not widely studied (10, 11). The aim of this study is to describe the antibiotic susceptibility pattern and the distribution of macrolide-resistance determinants of *S. pneumoniae* isolated from patients presented to Hospital Universiti Sains Malaysia (HUSM), Kelantan, Malaysia.

## Methods

### Pneumococcal Isolates

A total of 113 non-duplicate isolates of *S. pneumoniae* were collected from patients admitted to HUSM from June 2014 to December 2015. Pneumococcal isolates were collected from various clinical specimens including sputum, throat swab, endotracheal secretion, nasopharyngeal swab/aspirate, blood, cerebrospinal fluid (CSF), pus swab, high vaginal swab (HVS) and other body fluids. The isolates were presumptively identified as *S. pneumoniae* by their colony morphology, Gram-stain results and susceptibility to 5 μg optochin disc (ethyl hydrocuprein hydrochloride, Becton, Dickinson and Company, USA). The isolates were stored at –70 ^o^C in brain heart infusion broth plus 20% glycerol in the laboratory until further testing.

### Antimicrobial Susceptibility Test

The minimal inhibitory concentrations (MICs) of six antimicrobial agents: i) penicillin, ii) erythromycin, iii) azithromycin, iv) amoxicillin-clavulanic acid, v) trimethoprim-sulphamethoxazole and vi) vancomycin were determined by using E-test strips (BioMerieux SA, France) according to manufacturer’s recommendations. The MIC results are interpreted according to Clinical and Laboratory Standard Institute (CLSI) guidelines, 2015. *S. pneumoniae* ATCC 49619 was used as the control strain.

### Detection of Macrolide-Resistant Genes; erm(B) and mef(A) Genes

Out of 113 isolates of *S. pneumoniae*, 108 were subjected to polymerase chain reaction (PCR) analysis to determine the presence of macrolide-resistant genes. Three of the isolates were non-viable and the other two isolates were contaminated with other bacteria. According to the manufacturer’s instructions, DNA extractions were performed using a DNA extraction kit (DNeasy Blood & Tissue Kit, Qiagen, USA). The PCR mixture consists of a 20 μL total volume containing extracted DNA template, DreamTaq DNA polymerase (Thermo Scientific, Malaysia), 10X DreamTaq buffer which contains 20 mM MgCl2, 2 mM dNTP Mix, nuclease-free water and primer mixture. The primer sequences for *erm*(B) gene are 5′-CGTACCTTGGATATTCACCG-3′ and 5′-GTAAACAGTTGACGATATTCTCG-3′ whilst for *mef*(A) gene are 5′-CTGTATGGAGCTACCTGTCTGG-3′ and 5′-CCCAGCTTAGGTATACGTAC-3′ (12). The primer mixture was prepared by mixing an equal concentration of genes *erm*(B) and *mef*(A) primers (20 pmol/μL) for each forward and reverse primer. The amplification was performed with a thermal cycler (MJ Research Peltier Thermal Cycler, GMI, USA). The positive control strains; *S. pneumoniae* ATCC 700673 (Hungary19A-6) for *erm*(B) gene and *S. pneumoniae* ATCC 51916 (Tennessee23F-4) for *mef*(A) gene (13) and a negative control were included in each run. Following amplification, 2 μL of the PCR products were electrophoresed on 1% agarose gel and visualised using a UV light transilluminator (Syngene, USA) with GeneSnap image analysis software.

### Statistical Analysis

Statistical analysis was performed using SPSS, version 22.0. Fisher’s exact test or *X*^2^ test was applied to determine the association between the presence of macrolide resistance genes with mortality and complications.

## Results

### Distribution of Clinical Specimens of S. pneumoniae Isolates

A total of 113 non-duplicate isolates of *S. pneumoniae* from various clinical specimens were included in this study. The majority of the isolates were from sputum (33.6%), followed by endotracheal tube secretion (ETT) (29%), eye swab (14%), blood (10.6%), high vaginal swab (HVS) (2.7%), ear swab (1.8%) and corneal scrapping (1.8%). Other specimens (12.4%) included CSF, broncho-alveolar lavage (BAL) fluid, vitreous fluid, bone tissue, bile, nasal swab and pus swab.

### Demographic and Clinical Characteristics of Patients with S. pneumoniae Infection

Among the 113 patients, 22 (19.5%) were children aged 5 years old or below and 16 (14.2%) were elderly patients (≥ 65 years old). The median age of the patients was 33 years old. Of these patients, 73 (64.6%) were males and 40 (35.4%) were females. Community-acquired pneumonia was the most common type of infection (59.3%), followed by conjunctivitis (8.8%) and bacteremia (7.1%). About 9.9% of the patients had S. pneumoniae (the majority were from ETT specimens). The most common predisposing factors among patients with culture-positive *S. pneumoniae* were bronchopulmonary diseases (15.9%), underlying malignancy (6.2%) and chronic renal disease (4.4%).

### In vitro Susceptibility Pattern of S. pneumoniae Isolates

The susceptibility pattern of 113 *S. pneumoniae* isolates was shown in Table 1 and the MIC distribution of macrolide antibiotic based on the presence of macrolide resistance determinants was shown in Table 3.

### Distribution of erm(B) and mef(A) Genes

**Figure 1:**
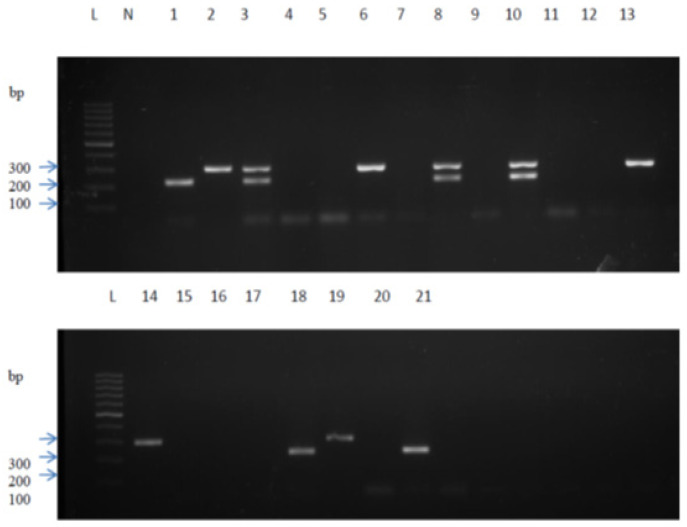
Scanned image of gel showing results of amplified DNA fragments of positive control (lanes 1–3) and 18 test isolates (lanes 4–21). PCR amplicons of *mef*(A) (298 bp) gene were shown in lanes 6, 13, 14 and 19, *erm*(B) (224 bp) gene in lanes 18 and 21 and a combination of *mef*(A) and *erm*(B) gene was shown in lane 8 and 10. A lane with no band indicates the absence of both genes Notes: Lane L = 100 bp DNA ladder; Lane N = negative control (DNase-free water); Lane 1 = positive control (*S. pneumoniae* ATCC 700673 (Hungary^19A^-6) - for *erm*(B) gene; Lane 2 = positive control (*S. pneumoniae* ATCC 51916) (Tennessee23F-4) - for *mef*(A) gene; Lane 3 = positive control (*S. pneumoniae* ATCC 700673 (Humgary^19A^-6) + *S. pneumoniae* ATCC 51916 (Tennessee^23F^-4) - for *erm*(B) gene + *mef*(A) gene combination; Lane 4–21 = *S. pneumoniae* isolates

Out of 108 isolates subjected to PCR analysis, *erm*(B) gene, *mef*(A) gene or a combination of *erm*(B) and *mef*(A) genes were identified in 31 (27.4%) isolates. Of these isolates, 20 (64.5%) had *mef*(A) gene alone and 6 (19.4%) had *erm*(B) gene alone, whereas 5 (16.15) contained both *mef*(A) and *erm*(B) genes. The PCR gel image showed amplified DNA fragments, as demonstrated in Figure 1.

Among 30 erythromycin-resistant *S. pneumoniae, mef*(A) and *erm*(B) genes were detected in 15 (50.0%) and 6 (20.0%) isolates, respectively. Both *mef*(A) and *erm*(B) genes were detected in 5 (16.7 %) isolates and 4 (13.3%) did not harbour either of the genes. Five (6.4%) of the erythromycin-sensitive isolates were detected to have have the *mef*(A) gene. Among the 34 azithromycin-resistant isolates, 15 (44.1%) and 5 (14.7%) had *mef*(A) gene and *erm*(B) gene, respectively, and 5 (14.7%) had both genes. Nine (26.5%) did not contain any of the genes. Two azithromycin-sensitive *S. pneumoniae* (10.0%) had *mef*(A) gene (Table 2). Isolates that harbour *erm*(B) gene alone or both *erm*(B) and *mef*(A) genes showed higher MICs ranging from 2 μg/mL to > 256 μg/mL and 8 μg/mL to > 256 μg/mL for erythromycin-resistant *S. pneumoniae* and azithromycin-resistant *S. pneumoniae*, respectively, as compared to isolates carrying *mef*(A) gene alone, with MICs ranging from 1 μg/mL to 32 μg/mL and 3 μg/mL to > 256 μg/mL for erythromycin-resistant *S. pneumoniae* and azithromycin-resistant *S. pneumoniae*, respectively (Table 3).

### Impact of Resistance to Mortality and Complications

Six patients (5.3%) died in the hospital while receiving treatment. One patient (0.9%) had empyema-related complications and another had subdural abscess-related complications. Two (1.8%) developed arrhythmia. The results (Tables 4 and 5) showed that there is no significant association between mortality rate and the development of complications between patients harbouring macrolide-resistance determinants with those without macrolide-resistance determinants.

## Discussion

In Malaysia, the prevalence of macrolide-resistant *S. pneumoniae* appeared to have increased over the years from 36.8% in 2001 (14) to 46.2% in 2005, then drastically increased to 62.2% in the year 2010 (15, 16). In our study, the rates of susceptibility towards macrolide antibiotics were not much different to those of a previous study (16), where the resistance rates to erythromycin and azithromycin were 26.5% and 30.1%, respectively. However, we reported a decrease in erythromycin resistance compared to more recent studies. However, the resistance rate towards erythromycin in 2010, which was more than 60%, was higher (14, 15).

The level of macrolide resistance has increased remarkably, with very high MIC_90_s value (64 μg/mL to ≥ 128 μg/mL) in Asian countries, including Malaysia (9). In this study, we found that isolates which are resistant to erythromycin or azithromycin also have high MIC values. The majority of them have an MIC of > 256 μg/mL, with MIC_90_ values of 32 μg/mL and 128 μg/mL for erythromycin and azithromycin, respectively. Compared with previous studies that investigated *S. pneumoniae* isolates in the years 2008–2010, the MIC_90_s of erythromycin are higher, with MIC values of ≥ 256 μg/mL (14, 15). In this study, 96.7% of erythromycin-resistant isolates were also resistant to azithromycin, which is consistent with previous studies where 94.4%–97% of the erythromycin-resistant pneumococcal isolates had concomitant erythromycin and azithromycin resistance (6, 15).

The *erm*(B) gene-mediated resistance in pneumococci gives high-level resistance (MLS_B_ phenotype), with MIC values typically ≥ 64 μg/mL. In contrast, the efflux pump (M phenotype) encoded by the *mef*(A) gene confers a low-level resistance, with MIC usually 1 μg/mL–32 μg/mL (9, 17, 18). Similarly, in this study, among the 30 erythromycin-resistant pneumococci, isolates that carry the *erm*(B) gene alone or both *erm*(B) and *mef*(A) genes showed higher MICs, ranging from 2 μg/mL to > 256 μg/mL, with MIC_90_ values for *erm*(B) gene being > 256 μg/mL. This is in contrast to isolates carrying *mef*(A) gene alone, which have much lower MICs ranging from 1 μg/mL to 32 μg/mL and a MIC_90_ of 4 μg/mL for mef(A) gene. These findings were in accordance with the other study that reported erythromycin-resistant *S. pneumoniae* isolates with both *erm*(B) and *mef*(A) genes showed very high MICs ≥ 256 μg/mL (14).

Ones risk of contracting pneumococcal infections varies depending on the age and gender of the individual. The prevalence of pneumococcal disease is substantially higher in children and the elderly above 65 years old than in young adults (2). The study’s findings also emphasise on the preponderance of pneumococcal disease among males, which might be linked to the higher incidence of underlying factors such as smoking and exposure to outdoor air pollution among males. Deficiencies in the non-specific or specific defence mechanisms against colonisation, aspiration or invasion of *S. pneumoniae* increase the likelihood of pneumococcal disease among this group of patients.

The clinical significance of the in-vitro resistance of macrolide antibiotics is still controversial (19). The failure of macrolide therapy caused by erythromycin-resistant strain in patients with pneumococcal diseases has been reported in previous studies (18, 20). A study investigating the mortality rates associated with invasive pneumococcal diseases reported almost similar findings with patients with erythromycin-resistant (18%) or erythromycin-susceptible strains (14%) (21). This study also had similar findings, where the mortality rate among patients with macrolide-resistant strains was not significantly different from that of patients with macrolide-susceptible strains (*P* > 0.999 for erythromycin and *P* = 0.667 for azithromycin). In this study, we also specifically assessed the relationship between the macrolide resistance determinants and the mortality and complications in patients with pneumococcal diseases since these resistant determinants are the main cause of macrolide resistance. This revealed a similar result where mortality and complication in patients carrying the resistance determinants did not differ from those in patients without resistance determinants. These findings were supported by another study by the Asian Network for Surveillance of Resistant Pathogens (ANSORP) group that reported that pneumococcal infection with erythromycin-resistant strains was not significantly associated with more severe illness or higher mortality (22).

The prevalence of the macrolide-resistant determinant gene of *S. pneumoniae* can provide the management teams in hospitals with crucial insights for making appropriate decisions on antimicrobial usage in patients’ treatment. By knowing its prevalence, healthcare professionals can understand the likelihood of resistance and choose alternative antibiotics for patients with macrolide-resistant infections, improving treatment efficacy. They may avoid unnecessary macrolide prescriptions in areas with high resistance rates, reducing the risk of further resistance development and preserving the effectiveness of these antibiotics. Furthermore, they can implement targeted infection control measures to limit the spread of macrolide-resistant infections within healthcare facilities.

This study has several limitations. The identification of *S. pneumoniae* isolates based on colony morphology, Gram-stain results and susceptibility to the optochin disc reflects the use of classical microbiological methods that have been historically relied upon for bacterial identification. These methods offer a cost-effective and relatively simple way to distinguish between different streptococcal species which is often encountered in clinical settings. However, the challenge arises because closely related species like *S. pseudopneumoniae* and *S. mitis* share similar characteristics and can be mistaken for *S. pneumoniae* using these conventional techniques. The similarity in optochin susceptibility and bile solubility between these species can lead to misclassification. As advancements in molecular techniques and genetic analysis have emerged, it has become apparent that incorporating more precise and specific methods, such as DNA sequencing and PCR-based assays, can enhance the accuracy of *S. pneumoniae* identification. However, due to limited resources and a small amount of grant money allocation, the molecular identification method could not be used.

Another limitation was the inadequacy of clinical data collected from medical records that largely depend on the clinician’s observation and interpretation. Incomplete documentation of the medical records will largely influence our data, especially on the predisposing factors and outcome of the diseases. Finally, since the sources of the isolates were collected only from the state of Kelantan, data from this study may not be representative of the Malaysian population.

## Conclusion

The majority of erythromycin-resistant isolates were found to have a *mef*(A) gene, an *erm*(B) gene or a combination of *mef*(A) and *erm*(B) genes. Overall, the isolates showed good susceptibility towards all antibiotics tested except for azithromycin. The outcome and complications of pneumococcal diseases were not significantly different between macrolide-resistant groups and macrolide-susceptible groups. They also were not affected by the presence of macrolide-resistant determinants in the pneumococcal isolates. Current data regarding the in-vitro susceptibility patterns of *S. pneumoniae* and the prevalence of the macrolide-resistant determinants gene could help the management team make appropriate decisions regarding antimicrobial usage in treating patients. Continuous surveillance of antibiotic resistance at the national level is very important to monitor the changing trends in antimicrobial resistance in Malaysia.

## Figures and Tables

**Table 1:** Susceptibility rates of *S. pneumoniae* isolate for six antimicrobial agents (*n* = 113)

Antibiotics	MIC_50_ (μg/mL)	MIC_90_ (μg/mL)	MIC range (μg/mL)	MIC interpretive criteria (μg/mL)			Antimicrobial susceptibility		

				S	I	R	S *n* (%)	I *n* (%)	R *n* (%)
Penicillin parenteral (nonmeningitis)	0.032	1	0.012–> 32	≤ 2	4	≥ 8	110 (97.3)	2 (1.8)	1 (0.9)
Penicillin parenteral (meningitis)	0.032	1	0.012–> 32	≤ 0.06	-	≥ 0.12	88 (77.9)	0 (0.0)	25 (22.1)
Penicillin (oral)	0.032	1	0.012–> 32	≤ 0.06	0.12–1	≥ 2	88 (77.9)	17 (15.0)	8 (7.1)
Erythromycin	0.125	32	0.03–> 256	≤ 0.25	0.5	≥ 1	83 (73.5)	0 (0.0)	30 (26.5)
Azithromycin	1	96	0.10–> 256	≤ 0.5	1	≥ 2	22 (19.5)	57 (50.4)	34 (30.1)
Amoxicillin-clavulanic acid	0.03	1	0.015–8	≤ 2/1	4/2	≥ 8/4	110 (97.3)	0 (0.0)	3 (2.7)
Trimethoprim-sulfamethoxazole	0.25	> 32	0.064–> 32	≥ 0.5/9.5	1/19–2/38	≥ 4/76	77 (68.1)	16 (14.2)	20 (17.7)
Vancomycin	0.5	0.5	0.12–1	≤ 1	-	-	113 (100.0)	0 (0.0)	0 (0.0)

Notes: MIC_50/90_ = MIC at which 50% or 90% of the isolates are inhibited; S = susceptible; I = intermediate; R= resistant

**Table 2:** Distribution of macrolide resistance determinants according to the susceptibility of macrolide antibiotics (*n* = 108)

Susceptibility to	No. of isolates with macrolide resistance determinant				Total *n* (%)

	*mef*(A) *n* (%)	*erm*(B) *n* (%)	*mef*(A) + *erm*(B) *n* (%)	*None* *n* (%)	
Erythromycin					
Sensitive	5 (6.4)	0 (0.0)	0 (0.0)	73 (93.6)	78 (100.0)
Intermediate	0 (0.0)	0 (0.0)	0 (0.0)	0 (0.0)	0 (0.0)
Resistant	15 (50.0)	6 (20.0)	5 (16.7)	4 (13.3)	30 (100.0)
Azithromycin					
Sensitive	2 (10.0)	0 (0.0)	0 (0.0)	18 (90.0)	20 (100.0)
Intermediate	3 (5.6)	1 (1.9)	0 (0.0)	50 (92.6)	54 (100.0)
Resistant	15 (44.1)	5 (14.7)	5 (14.7)	9 (26.5)	34 (100.0)

**Table 3:** MIC distribution of macrolide antibiotic based on the presence of macrolide resistance determinant (*n* = 113)

Antimicrobial agent	MIC (μg/mL) distribution																		MIC range

	0.032	0.125	0.25	0.5	0.75	1	2	3	4	6	8	12	16	24	32	48	96	> 256	
Erythromycin^a^																			
*erm*(B)													1					5	16–> 256
*mef*(A)	1	3	1			1	2	1	3		5		1	1	1				0.032–> 32
e*rm*(B) + m*ef*(A)							1											4	2–> 256
Azithromycin^b^																			
*erm*(B)						1												5	1–> 256
m*ef*(A)		1		1	2	1		1	2	2	1	2	1		1	1	3	1	0.125–> 256
e*rm*(B) + m*ef*(A)											1							4	8–> 256

Notes:

a Resistance to erythromycin was defined as MIC ≥ 1 μg/mL;

b Resistance to azithromycin was defined as MIC ≥ 2 μg/mL

**Table 4:** Association between macrolide resistance determinants with clinical outcome (*n* = 111)

Macrolide resistance determinant	Outcome		*P*-value^a^

	Alive *n* (%)	Death *n* (%)	
*erm*(B)	6 (100)	0 (0)	0.837
*mef*(A)	18 (90)	2 (10)	
*erm*(B) + m*ef*(A)	4 (100)	0 (0)	
None	72 (94.7)	4 (5.3)	
Non-viable/contaminated	5 (100)	0 (0)	

Note:

a Fisher’s exact test was applied

**Table 5:** Association between macrolide resistance determinants with complications (*n* = 111)

Complication	Genes						*P*-value^a^

		*erm*(B)	*mef*(A)	*erm*(B) + *mef*(A)	None	NV/C	
Empyema	Absent	6 (100)	20 (100)	4 (100)	75 (98.7)	5 (100)	> 0.999
	Present	0 (0)	0 (0)	0 (0)	1 (1.3)	0 (0)	
Subdural abscess	Absent	5 (83.3)	20 (100)	4 (100)	76 (100)	5 (100)	0.135
	Present	1 (16.7)	0 (0)	0 (0)	0 (0)	0 (0)	
Cardiac	Absent	6 (100)	20 (100)	4 (100)	74 (97.4)	5 (100)	> 0.999
	Present	0 (0)	0 (0)	0 (0)	2 (2.6)	0 (0)	

Notes:

a Fisher’s exact test was applied; NV/C= non-viable/contaminated
